# Patient-Specific Instrumentation vs Standard Referencing in Total Ankle Arthroplasty: A Comparison of the Radiologic Outcome

**DOI:** 10.1177/10711007221077100

**Published:** 2022-02-24

**Authors:** Lukas Heisler, Werner Vach, Georg Katz, Thomas Egelhof, Markus Knupp

**Affiliations:** 1 University of Basel, Basel, Switzerland; 2 Basel Academy for Quality and Research in Medicine, Basel, Switzerland; 3 Merian Iselin Klinik, Institute for Radiology, Basel, Switzerland; 4 Orthopedic Surgery & Traumatology, Mein Fusszentrum AG, Basel, Switzerland

**Keywords:** total ankle arthroplasty, patient specific instrumentation, ankle replacement, ankle arthritis, ankle prothesis

## Abstract

**Background:**

Existing literature on the superiority of patient-specific instrumentation (PSI) in total ankle arthroplasty (TAA) over standard referencing (SR) is limited. Advantages presented include better implant alignment, shorter operating times, and increased accuracy of implant size prediction. The aim of this retrospective study was to analyze PSI in the hands of an experienced foot and ankle surgeon new to both PSI and SR for this specific implant, in regard to determining implant alignment, operative times, and radiologic short-term outcome and predicting implant size for tibial and talar components.

**Methods:**

Twenty-four patients undergoing TAA using PSI were compared to 25 patients using SR instrumentation. Outcome measures included alignment of the tibial component (α coronal plane, γ sagittal plane), the tibiotalar tilt (β), and the talar offset x on the sagittal view as well as the presence of radiolucent lines, operation time, and wound healing. Postoperative outcome was assessed at 6 weeks, 4 months, and 1 year postoperatively.

**Results:**

Implant positioning was similar in both groups, and no advantage in regard to the operative time could be seen when comparing TAA using PSI to SR. Implant size prediction was more reliable for the tibia than for the talus. Three patients (1 from the SR group and 2 from the PSI group) showed radiolucent lines around the tibial component. Two patients (both SR group) suffered delayed wound healing, albeit not requiring any additional measures.

**Conclusion:**

The PSI method did not show an advantage over SR in regard to positioning of the components or the duration of the surgery. The current study suggests that no initial advantage of PSI over SR are to be expected in standard total ankle replacement.

**Level of Evidence:**

Level III, retrospective study.

## Introduction

Total ankle arthroplasty (TAA) has evolved into an established alternative to ankle arthrodesis in end-stage ankle arthritis. Preserving joint mobility is thought to lead to a more physiological gait pattern and thereby reducing the risk for adjacent joint arthritis when compared to ankle arthrodesis.^17,20^ However, comparative data show a higher risk for revisions and complications in arthroplasty than in ankle arthrodesis.^8^ Although infections and wound breakdown are the most common early reasons for failure in TAA,^14^ malalignment of the implants is one of the main risk factors for failure in the long term.^12^

In TAA, intraoperative alignment control of component positioning is done by standard referencing (SR) with the jigs provided by the implant suppliers, navigation or with patient-specific instrumentation (PSI). Although navigation in ankle replacement so far has proven to be difficult because of the lack of reliable landmarks around the ankle joint, many surgeons implemented PSI for their total ankle replacements.^6,11^ However, current literature is limited and controversially discusses the advantage of PSI over SR. Some aspects considered include the higher accuracy of especially tibial implant positioning in PSI over SR and shorter operative times, which coincide with a reduced risk for wound healing disorders in PSI.^2,6,9,11,13,18,19^ Disadvantages of PSI include the higher costs, the need for extensile periosteum stripping during surgery, and the need for a preoperative computed tomographic scan.^11^

The aim of this study was to compare a homogenic patient cohort treated for ankle arthritis with TAA by a senior surgeon, who was new to the use of PSI and SR for this specific implant in regard to the accuracy of both tibial and talar implant positioning, the presence of radiolucent lines on postoperative radiographs to determine the rate of delayed osteointegration/radiolucent lines, and operative times and wound healing problems. Our hypothesis was that PSI would lead to higher accuracy of implant positioning for both the tibial and talar side, shorter operative times, and accurate implant size prediction.

## Methods

### Study Design

A consecutive series of patients with end-stage osteoarthritis who had received a primary total ankle replacement type Infinity (Wright Medical Technology) as an isolated bony procedure between August 2018 and December 2019 and had signed an informed consent were eligible for enrollment. All replacements have been implanted by a single surgeon who was a new user of the Infinity ankle system and had no experience with the use of PSI. The surgeon was a fellowship-trained surgeon who had implanted ankle replacement for more than 18 years. Exclusion criteria included additional bony procedures (osteotomies and/or adjacent joint fusions, 11 cases), patients who received an implant from another manufacturer, revision arthroplasty, and patients with previous ankle fusions (take down procedures). Patient and management characteristics are shown in Table 1. The patients in each group were assessed clinically and radiographically 1 year postoperatively by 2 independent assessors (L.H. and G.K.). The protocol was approved by the ethical committee (Ethik Kommission Nordwest- und Zentralschweiz, reference number 2020-02806) and is in accordance with the ethical standards of the Declaration of Helsinki and with the Guidelines for Good Clinical Practice. All surgeries were performed by the senior author (M.K.).

**Table 1.** Patient and Management Characteristics of Both SR and PSI groups.^a^

	SR	PSI
Age, mean ± SD	66.1 ± 10.7	60.7 ± 10.3
Male	15 (60)	19 (79)
Right side	12 (48)	15 (63)
Additional soft tissue procedures	3 (12)	8 (33)
>10-degree deviation in at least 1 angle	9 (36)	8 (33)

Abbreviations: PSI, patient-specific instrumentation; SR, standard referencing.

a Values shown are absolute (relative) frequencies or mean ± SD.

The 2 groups were compared with respect to the accuracy of the radiologic implant alignment, the occurrence of radiolucent lines, the operative time, the occurrence of postoperative wound healing disturbances, and the reliability of the preoperative protocol with respect to predicting the implant sizes.

### Radiographic Analysis

Standard weightbearing anteroposterior and lateral radiographs of the ankle and dorsoplantar views of the foot were taken preoperatively, 6 weeks, 16 weeks, and 1 year postoperatively in all patients as part of their standard clinical care. The radiographs at 6 weeks and at 16 weeks were used to determine the achieved alignment. The postoperative images with the best quality were used to measure the angles. The presence of radiolucent lines was evaluated at 16 weeks and 1 year postoperation. All radiographic outcomes were reassessed as part of this study by a fellowship-trained senior musculoskeletal radiologist (G.K.) and a PhD student (L.H.). Both were instructed by the senior author using radiographs not involved in this study. They were anonymized for each other and any clinical information about the patients, in particular with respect to the referencing method used. The measurements of the 2 assessors were compared to assess interobserver reliability, and a low interobserver variability was found.

**Figure 1.**
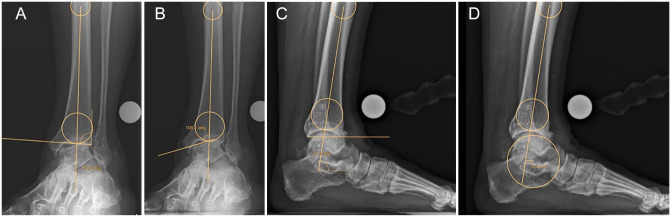
Illustration of the (A) preoperative angle α; (B) preoperative angle β; (C) preoperative angle γ; and (D) preoperative talar offset with respect to the tibial axis.

**Figure 2.**
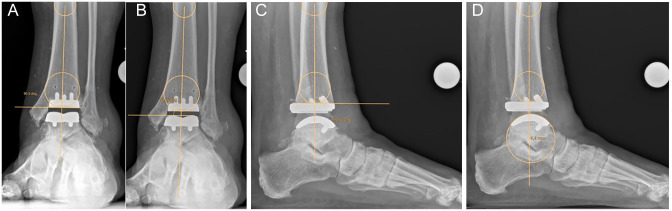
Illustration of the (A) postoperative angle α; (B) postoperative angle β; (C) postoperative angle γ; and (D) postoperative talar offset with respect to the tibial axis.

Assessment included 2 angles on the coronal plane (α and β, Figure 1A, B) and 1 angle and 1 distance on the sagittal plane (γ and *x*, Figure 1C, D). The angle α was measured between the axis of the tibia (midpoint of the tibia at 2 evenly spaced intervals on the anteroposterior ankle view) and the joint line. The angle β was measured between the axis of the tibia and the talar surface. The angle γ was measured between the axis of the tibia and the distal tibial joint line. The distance *x* was defined as the orthogonal offset of the talar center relative to the axis of the tibia, an anterior offset received positive values and posterior offset negative values. Postoperatively, the joint line for α, β, and γ was defined as the tibial implant and the offset (*x*) measured to the center of the talar implant (Figure 2). These measurements are similar to those used in previously published literature.^19^

The patients who underwent a TAA using PSI received, in addition to the standard radiographs, a standard computed tomographic scan from the knee through the midfoot according to a protocol developed by the implant manufacturer. From these scans, a 3-dimensional bone model was made and used to determine anatomic reference points on the tibia and talus.

**Figure 3.**
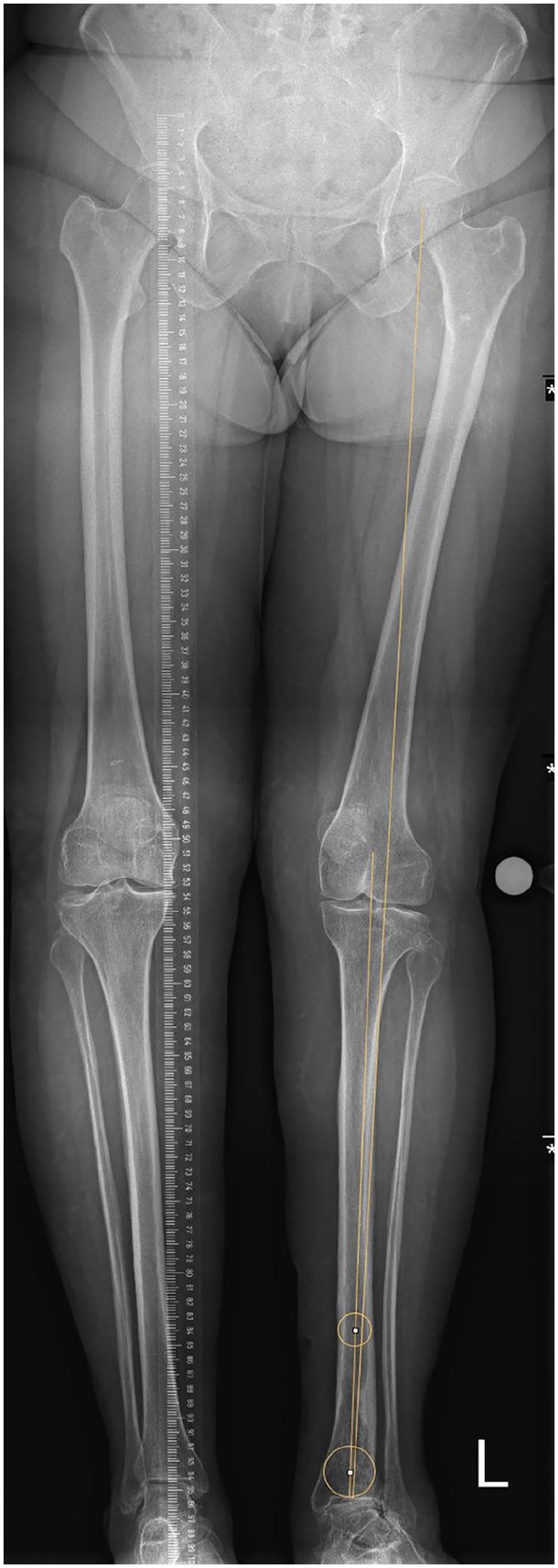
Illustration of the deviation of the mechanical from the anatomical axis.

In order to take into account that PSI aimed at placing the implant in an optimal manner relative to the mechanical axis, the angle describing the difference between the mechanical axis and the anatomical axis was taken into account (Figure 3): this value could be extracted from the PSI documentation in each patient. For patients in the PSI group, these differences in the coronal plane were added to the angles α and β, if the mechanical axis was lateral to the anatomical axis and subtracted otherwise. The difference in the sagittal plane was added to the angle γ, if the mechanical axis was posterior to the anatomical axis and subtracted otherwise.

Presence of radiolucent lines was defined as any radiolucency greater than 2 mm observed in one of the radiologic images taken 6 weeks, 16 weeks, or 1 year after TAA.

### Preoperative Plan

Besides the radiographic analysis, the preoperative report was assessed for the patients treated with PSI. The predicted tibial and talar implant sizes were compared to the actual size implanted.

### Clinical Follow-up

All patients participated in clinical follow up visits after 6 weeks, 16 weeks, and 1 year. The presence of any wound infection during the 1-year follow-up period was extracted from the medical records.

### Duration of Operation

Duration of operation was extracted from the surgeons’ report.

### Statistical Analysis

The interobserver reproducibility of the single angles and offsets were described by the median of the absolute deviations and the intraclass correlation coefficient. For further analyses, the average over the 2 replicates was used.

The distribution of continuous variables is described by means and standard deviations, except for variables for which a skewed distribution was expected (in particular, the absolute deviations from the intended alignment). In these variables we reported the median and the 90th percentile. Further distributional characteristics of measured and derived variables are reported in Supplementary Table 1. The distribution of binary and categorical variables is described by absolute and relative frequencies.

The primary outcome are the absolute deviations from the intended alignment of 90 degrees for the 3 angles α, β, and γ. Their distribution in each patient group is depicted by histograms. The statistical significance of a difference between the 2 groups across all 3 angles was assessed by a multivariate analysis of variance. The absolute deviations were categorized into 3 groups using cut points of 3 degrees and 5 degrees as suggested by Saito et al.^19^

The deviations themselves served as a secondary outcome. A multivariate analysis of variance was used to assess the significance of a deviation of the mean deviations from 0 degrees within each patient group as well as the difference between the 2 groups.

The joint distribution of the measured angles underlying the deviations was visualized by pairwise scatterplots. The same technique was used to visualize the distribution of the preoperative angles.

Further secondary outcomes were the offset *x*, the occurrence of radiolucency, the duration of surgery, and the occurrence of wound infections. To assess the statistical significance of the difference between the 2 groups, the Wilcoxon test or Fisher exact test was used. For the duration, we additionally performed an analysis adjusted for the presence of additional soft tissue procedures (lateral ligament reconstructions, deltoid ligament release, peroneal tendon repairs), as the latter was more frequent in the PSI group.

The reliability of the implant size prediction was assessed by a cross-tabulation of the predicted and the actual implant size in the patients in the PSI group.

### Sample Size Calculation

With respect to the absolute deviation from the intended alignment, Saito et al^19^ reported population standard deviations between 1.2 and 1.5 degrees for the angles α and γ. Assuming a standard deviation of 1.35 degrees, we would have a power of 80% to detect a mean difference between the 2 patient groups in absolute angle values of 1 degree by a Student *t* test at the 5% level. However, we summarized the statistical evidence for a group difference across all 3 angles in a single *P* value, which increases the power.

## Results

Forty-nine patients were eligible for the study. Twenty-five TAAs were performed with SR and 24 with PSI. No patient was lost to follow-up. Basic patient characteristics are shown in Table 1. The patient groups were comparable with respect to age, gender, and laterality. However, patients in the PSI group tended to have additional soft tissue procedures performed more often as part of the surgical intervention.

### Interobserver Variability

In the assessment of the postoperative images, the median absolute differences between the 2 observers were 0.6, 0.7, and 0.4 degrees for alpha, beta, and gamma, respectively, and 0.7 mm for the offset. The corresponding intraclass correlation coefficient values were all 0.89 or above. The observer variability was distinctly larger in assessing the preoperative images. Additional details are given in Supplementary Table 2.

### Preoperative Values

**Figure 4.**
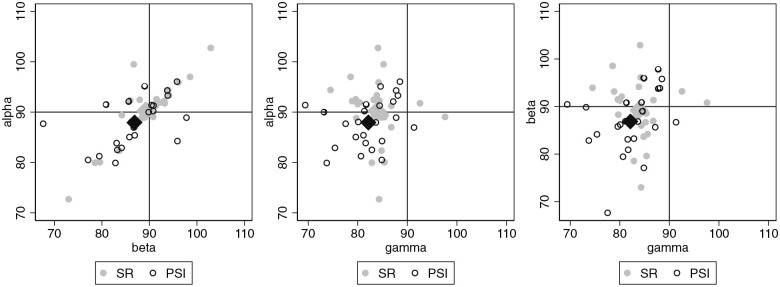
Preoperative values of the 3 angles alpha, beta, and gamma visualized by pairwise scatter plots. The group-specific mean values are shown as diamonds.

The joint distribution of the preoperative values of the 3 primary outcomes is depicted in Figure 4 by pairwise scatterplots. The 2 patient groups show very similar distributions. The median / 90th percentiles of the offset *x* were 4.7/6.8 in the SR group and 6.3/13.4 in the PSI group, pointing to greater preoperative offsets, and thereby more challenging positioning of the implants, in the PSI group. The absolute deviations between the mechanical and anatomical axis in the PSI group showed a median of 1.15 degrees both in the coronal and sagittal plane and 90% percentiles of 3 and 2.9 degrees, respectively.

### Postoperative Alignment

**Figure 5.**
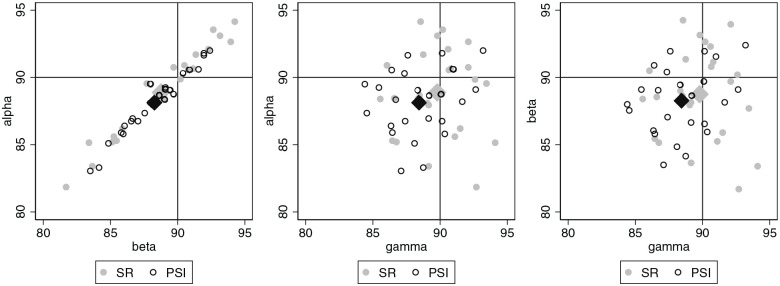
Postoperative values of the 3 angles alpha, beta, and gamma visualized by pairwise scatter plots. The group-specific mean values are shown as diamonds.

The distributions of the postoperative values (Figure 5) indicate that for most patients it was possible to approach the intended angles and that the angles α and β tend to be close together, indicating successful correction in tilted ankles.

The distribution of the (absolute) deviations from the intended alignment are shown in Table 2. The distributions were very similar between the 2 groups. However, when taking the sign of the deviation into account (Table 3), the deviation was more pronounced in the PSI group, reaching a significant difference.

**Table 2.** Absolute Deviations From the Intended Alignment.^a^

	Alpha	Beta	Gamma	*P* value
SR	2.1/4.9	2.3/6.3	1.5/3.9	.624
PSI	1.8/4.9	2.0/5.2	2.5/4.5	

Abbreviations: PSI, patient-specific instrumentation; SR, standard referencing.

a Values shown are medians and 90th percentiles and the *P* value for a difference between the 2 groups.

**Table 3.** Absolute Deviations From the Intended Alignment Classified Into Groups.^a^

	<3 degrees	3-5 degrees	>5 degrees
Alpha			
SR	15 (60)	8 (32)	2 (8)
PSI	15 (62)	7 (29)	2 (8)
Beta			
SR	15 (60)	7 (28)	3 (12)
PSI	16 (67)	5 (21)	3 (12)
Gamma			
SR	18 (72)	7 (28)	0 (0)
PSI	16 (67)	6 (25)	2 (8)

Abbreviations: PSI, patient-specific instrumentation; SR, standard referencing.

a Values shown are absolute frequencies, with relative frequencies in parentheses.

### Secondary Outcomes

Table 4 depicts further secondary outcomes. The distribution of the postoperative offset x was similar in both groups both when considering the measured values as well as the absolute deviations from 0. Radiolucency was observed in one patient in the SR group and in 2 patients in the PSI group. One wound infection was observed in the SR group. The average operation duration was 94 minutes in the SR group and 110 minutes in the PSI group. Even if we adjusted the operative time by taking the additional soft-tissue procedures into account, there remained a significant increase in the PSI group of 13 minutes (*P* = .006).

**Table 4.** Distribution of Secondary Outcomes.

	SR	PSI	*P* Value
Offset, mean±SD	2.3±2.0	2.6±2.8	.631
Absolute offset, median/p90	3.1/4.1	2.9/6.2	.480
Radiolucency^a^	1 (4)	2 (8)	.609
Wound infections^a^	1 (4)	0 (0)	>.999
Duration in minutes, mean ± SD	93.7±13.7	110.3±19.2	.005

Abbreviations: PSI, patient-specific instrumentation; p90, 90th percentile; SR, standard referencing.

a Values are absolute frequencies, with relative frequencies in parentheses.

### Reliability of the Implant Size Predicted by PSI

With respect to the tibial component, the prediction of the implant size was correct in 88% of the patients (21/24 cases). With respect to the talus component, PSI was correct in two-thirds of the patients (16/24 cases) and mainly overestimated the size in the remaining patients, especially in patients requiring a size of 4.

## Discussion

Our data suggest no difference in the radiologic outcome between the 2 techniques in standard isolated TAA. Wound infections and radiolucent lines appeared at such a low frequency that no conclusion about group differences can be made.

TAA has become a reliable alternative to ankle arthrodesis in patients with end-stage osteoarthritis.^1,4,5,7,16,21^ Although in ball and socket joints (hip and shoulder), implant malpositioning in any direction can be compensated for to a certain extent, this is only possible to a very limited amount in the knee and ankle joint. Therefore, longevity of an ankle replacement is tightly related to correct positioning of the implants.^12^ Intraoperative referencing in the knee and hip by navigation have gained increasing popularity; however, this technique is not available for ankle replacement. The surgeon must rely on the jigs provided by the manufacturer of the implant (SR) or on PSI. In contrast to the knee, standard referencing is not possible by intramedullary devices (to our knowledge, only 1 implant provides intramedullary devices) but solely relies on external landmarks, that is, the tibial tuberosity. Therefore, several companies implemented the option of PSI for their ankle replacement systems. In our study, we sought to assess the advantages of PSI in the hands of an experienced foot and ankle surgeon new to this implant, with respect to the achieved alignment, osteointegration of the implants, decreased operative time and wound healing disturbances and the reliability of the preoperatively predicted implant size for both the tibial and talar implant. In our study, the senior author changed to the Infinity total ankle system for primary total ankle replacement in 2018, and we compared the first 25 cases of each technique for an isolated primary total ankle replacement.

When comparing the 2 groups in terms of implant alignment, we did not find any difference in the accuracy of implant positioning. This is in line with the results of Saito et al,^18,19^ who reported very similar mean values for the absolute deviation from the intended alignment for the angles α and β. However, they obtained overall a distinctly higher accuracy, reaching deviations of less than 3 degrees in more than 80% of their patients.^19^

The advantages of using PSI have been evaluated in several studies so far.^2,6,9,11,13,19^ Two studies^6,13^ investigated the accuracy of the final alignment when using PSI, without comparing to SR. Two further studies^11,19^ presented a comparison with SR. Both studies concluded a similar accuracy between the techniques, although in the study of Hamid et al^11^ the alignment was worse in the PSI group. However, both studies included patients with many additional procedures performed during surgery. In the current study, patients with additional bony procedures were excluded, resulting in a more homogenic patient cohort. To focus on the impact of using PSI or SRI on the performance of TAA, we excluded patients with additional bony procedures from the study. This should not be misunderstood in thinking that PSI should not or need not to be combined with additional bony procedures. This was done to keep the groups more homogenic. In general, the majority of severe valgus / varus ankle arthritis cases will require additional bony procedures in addition to soft tissue balancing. In addition to the previously analyzed positioning of the tibial component, we assessed the position of the talar component. On the anteroposterior view, tilted ankles showed normalization in both groups. This was also found for the assessment in the sagittal plane, were the talar offset in relation to the tibia was reduced on average in both groups.

### Radiolucency

The use of PSI requires extensile periosteum stripping to guarantee adequate bony contact of the customized guides. This is of concern because it may carry the risk of impaired bony ingrowth of the implants. Escudero et al found a slightly higher, though not significant, risk of osteolysis in the PSI group compared with SR.^10^ This agrees with our findings, wherein a trend for a higher risk for radiolucent lines was observed in the PSI group (2 vs 1 case in the SR group). However, the numbers in the current study are too low to conclude whether the increased intraoperative damage to the periarticular bone affects the incorporation of the implants in TAA, and more research is needed before a conclusion can be drawn.

### Preoperative Plan

The preoperative plan predicted the tibial size in 21 of 24 cases and the talar size in 16 of 24 cases. These numbers are slightly better than reported by Saito et al (tibia 55 / 75 cases and 38 / 75 cases).^19^ The preoperative plan tends to overestimate the implant size, particularly on the talar side. This observation is in accordance with others.^6,19^

### Operative Time

We did not find a superiority of PSI compared to the SR method when considering the operative time. This is in contrast with earlier reports^11,18,19^ and may have been caused by the patient selection criteria: cases that were considered challenging because of severe malalignment, altered ligamentous status, or impaired bone stock were more often treated with PSI. Furthermore, we report on the surgeons’ first 25 cases using the PSI system and getting used to the technique may have led to prolonged operative times.

### Limitations

The main limitation of this study was the method of the radiologic assessment of the ankle joints. The authors followed the principles established in previous studies. As the shape of the tibia varies considerably, assessment of angles around the ankle joint on ankle radiographs does not seem very reliable. Ideally, the alignment should be assessed on images including the ankle, the knee, and the hip joint.^3^ This is not the case in routine radiographs, leading to the necessity to perform a post hoc correction in the PSI group as was done by Saito et al.^19^ However, the assessment of the radiologic parameters themselves remains a manual process and is affected by observer variation. The 2 observers involved in this study showed a sufficiently low interobserver variability to be able to regard our measurements on the postsurgical images as reliable. Second, one of the great advantages of PSI may be the determination of the rotation of the implant.^15^ This parameter was not assessed in our study.

Furthermore, the cohort assessed in the current study included the first patients receiving an Infinity TAA by the senior author. It has been postulated that PSI supports the surgeon in becoming familiar with a new implant. This would imply higher accuracy of the implant positioning in the PSI group, which, however, was not observed in the current study.

Additionally, the patients in this cohort were not randomized. Radiographic parameters measured on the preoperative radiographs retrospectively showed a trend that challenging cases were more often treated with PSI and straightforward cases with SR. Last but not least, the extra costs of PSI are not covered by health insurance in our country, unless the patients have private insurance. Therefore, the latter group was more likely to have access to the PSI method. However, at the end, it was the surgeon’s choice as to which method was applied.

A major advantage of our study is the exclusion of patients with additional bony procedures to keep the group as homogenic as possible, which has not been applied in previous studies so far. Nonetheless, a definitive statement about the group differences in the occurrence of radiolucent lines or wound infections was not possible owing to the limited sample size. The observed frequencies in the magnitude of 5% to 10% suggest that this may be a clinically relevant issue. There is thus a need for large-scale multicenter studies or registries to address this question.

## Conclusions

Our data suggest that patient-specific instrumentation does not yet provide any meaningful initial advantage in the standard ankle replacement when it comes to the accuracy of coronal or sagittal positioning for both tibial and talar component positioning. This is also the case for implant size estimation and operative time. Furthermore, we observed—in line with a previous study—a trend to worse osteointegration/radiolucent lines. However, further research needs to be done to evaluate whether this can be corroborated in a bigger cohort and for a longer follow-up.

## Supplemental Material

sj-docx-1-fai-10.1177_10711007221077100 – Supplemental material for Patient-Specific Instrumentation vs Standard Referencing in Total Ankle Arthroplasty: A Comparison of the Radiologic Outcome Click here for additional data file. Supplemental material, sj-docx-1-fai-10.1177_10711007221077100 for Patient-Specific Instrumentation vs Standard Referencing in Total Ankle Arthroplasty: A Comparison of the Radiologic Outcome by Lukas Heisler, Werner Vach, Georg Katz, Thomas Egelhof and Markus Knupp in Foot & Ankle International

sj-docx-2-fai-10.1177_10711007221077100 – Supplemental material for Patient-Specific Instrumentation vs Standard Referencing in Total Ankle Arthroplasty: A Comparison of the Radiologic Outcome Click here for additional data file. Supplemental material, sj-docx-2-fai-10.1177_10711007221077100 for Patient-Specific Instrumentation vs Standard Referencing in Total Ankle Arthroplasty: A Comparison of the Radiologic Outcome by Lukas Heisler, Werner Vach, Georg Katz, Thomas Egelhof and Markus Knupp in Foot & Ankle International

sj-docx-3-fai-10.1177_10711007221077100 – Supplemental material for Patient-Specific Instrumentation vs Standard Referencing in Total Ankle Arthroplasty: A Comparison of the Radiologic Outcome Click here for additional data file. Supplemental material, sj-docx-3-fai-10.1177_10711007221077100 for Patient-Specific Instrumentation vs Standard Referencing in Total Ankle Arthroplasty: A Comparison of the Radiologic Outcome by Lukas Heisler, Werner Vach, Georg Katz, Thomas Egelhof and Markus Knupp in Foot & Ankle International

sj-docx-4-fai-10.1177_10711007221077100 – Supplemental material for Patient-Specific Instrumentation vs Standard Referencing in Total Ankle Arthroplasty: A Comparison of the Radiologic Outcome Click here for additional data file. Supplemental material, sj-docx-4-fai-10.1177_10711007221077100 for Patient-Specific Instrumentation vs Standard Referencing in Total Ankle Arthroplasty: A Comparison of the Radiologic Outcome by Lukas Heisler, Werner Vach, Georg Katz, Thomas Egelhof and Markus Knupp in Foot & Ankle International

sj-pdf-5-fai-10.1177_10711007221077100 – Supplemental material for Patient-Specific Instrumentation vs Standard Referencing in Total Ankle Arthroplasty: A Comparison of the Radiologic Outcome Click here for additional data file. Supplemental material, sj-pdf-5-fai-10.1177_10711007221077100 for Patient-Specific Instrumentation vs Standard Referencing in Total Ankle Arthroplasty: A Comparison of the Radiologic Outcome by Lukas Heisler, Werner Vach, Georg Katz, Thomas Egelhof and Markus Knupp in Foot & Ankle International
