# Supplementary material for: Patient-Specific Instrumentation vs Standard Referencing in Total Ankle Arthroplasty: A Comparison of the Radiologic Outcome
Source: Foot Ankle Int. 2022 Feb 24;43(6):741–9. doi: 10.1177/10711007221077100 (PMC9168897; doi:10.1177/10711007221077100)
Supplement: sj-docx-1-fai-10.1177_10711007221077100 – Supplemental material for Patient-Specific Instrumentation vs Standard Referencing in Total Ankle Arthroplasty: A Comparison of the Radiologic Outcome [file sj-docx-1-fai-10.1177_10711007221077100.docx]

Supplementary Table 1: Distributional characteristics of measured and derived variables

| Preoperatively measured values | | | | | | | | | |
| --- | --- | --- | --- | --- | --- | --- | --- | --- | --- |
| group | var | N | mean | sd | median | p10 | p90 | min | max |
| SR | alpha | 25 | 90.2 | 6.4 | 91.6 | 80.1 | 97.0 | 72.7 | 102.8 |
| PSI | alpha | 24 | 88.8 | 4.4 | 89.1 | 83.9 | 94.8 | 81.8 | 97.2 |
| SR | beta | 25 | 89.2 | 6.4 | 89.3 | 79.6 | 96.1 | 73.0 | 102.9 |
| PSI | beta | 24 | 87.8 | 6.8 | 87.1 | 81.9 | 96.9 | 67.1 | 99.8 |
| SR | gamma | 25 | 83.9 | 4.6 | 84.2 | 78.8 | 86.8 | 74.5 | 97.6 |
| PSI | gamma | 24 | 82.4 | 5.7 | 83.2 | 74.0 | 89.0 | 69.9 | 90.3 |
| SR | offset | 25 | 4.1 | 4.1 | 4.7 | 0.3 | 6.8 | -10.3 | 13.4 |
| PSI | offset | 24 | 5.8 | 6.1 | 6.3 | -1.9 | 13.4 | -5.2 | 18.5 |

| Preoperatively measured values after correction in PSI group | | | | | | | | | |
| --- | --- | --- | --- | --- | --- | --- | --- | --- | --- |
| group | var | N | mean | sd | median | p10 | p90 | min | max |
| SR | alpha | 25 | 90.2 | 6.4 | 91.6 | 80.1 | 97.0 | 72.7 | 102.8 |
| PSI | alpha | 24 | 87.9 | 4.7 | 88.0 | 81.2 | 94.3 | 79.9 | 96.1 |
| SR | beta | 25 | 89.2 | 6.4 | 89.3 | 79.6 | 96.1 | 73.0 | 102.9 |
| PSI | beta | 24 | 86.9 | 6.7 | 86.8 | 79.5 | 95.8 | 67.7 | 97.8 |
| SR | gamma | 25 | 83.9 | 4.6 | 84.2 | 78.8 | 86.8 | 74.5 | 97.6 |
| PSI | gamma | 24 | 82.1 | 5.4 | 82.3 | 73.8 | 88.1 | 69.4 | 91.3 |

| Preoperative deviations | | | | | | | | | |
| --- | --- | --- | --- | --- | --- | --- | --- | --- | --- |
| group | variable | N | mean | sd | median | p10 | p90 | min | max |
| SR | alpha | 25 | 0.2 | 6.4 | 1.6 | -9.9 | 7.0 | -17.3 | 12.8 |
| PSI | alpha | 24 | -1.1 | 4.4 | -1.0 | -6.1 | 4.8 | -8.2 | 7.2 |
| SR | beta | 25 | -0.8 | 6.4 | -0.7 | -10.4 | 6.1 | -17.0 | 12.9 |
| PSI | beta | 24 | -2.2 | 6.8 | -2.9 | -8.1 | 6.9 | -22.9 | 9.8 |
| SR | gamma | 25 | -6.1 | 4.6 | -5.8 | -11.2 | -3.2 | -15.5 | 7.6 |
| PSI | gamma | 24 | -7.6 | 5.7 | -6.8 | -16.0 | -1.0 | -20.1 | 0.3 |

| Preoperative deviations after correction in PSI group | | | | | | | | | |
| --- | --- | --- | --- | --- | --- | --- | --- | --- | --- |
| group | variable | N | mean | sd | median | p10 | p90 | min | max |
| SR | alpha | 25 | 0.2 | 6.4 | 1.6 | -9.9 | 7.0 | -17.3 | 12.8 |
| PSI | alpha | 24 | -2.1 | 4.7 | -2.0 | -8.8 | 4.3 | -10.1 | 6.1 |
| SR | beta | 25 | -0.8 | 6.4 | -0.7 | -10.4 | 6.1 | -17.0 | 12.9 |
| PSI | beta | 24 | -3.1 | 6.7 | -3.2 | -10.5 | 5.8 | -22.3 | 7.8 |
| SR | gamma | 25 | -6.1 | 4.6 | -5.8 | -11.2 | -3.2 | -15.5 | 7.6 |
| PSI | gamma | 24 | -7.9 | 5.4 | -7.7 | -16.2 | -1.9 | -20.6 | 1.3 |

| Preoperative absolute deviations | | | | | | | | | |
| --- | --- | --- | --- | --- | --- | --- | --- | --- | --- |
| group | variable | N | mean | sd | median | p10 | p90 | min | max |
| SR | alpha | 25 | 4.5 | 4.4 | 2.4 | 0.9 | 10.1 | 0.6 | 17.3 |
| PSI | alpha | 24 | 3.8 | 2.5 | 3.9 | 0.7 | 7.2 | 0.4 | 8.2 |
| SR | beta | 25 | 4.7 | 4.3 | 3.2 | 0.9 | 11.4 | 0.7 | 17.0 |
| PSI | beta | 24 | 5.3 | 4.6 | 4.8 | 1.3 | 9.3 | 0.8 | 22.9 |
| SR | gamma | 25 | 7.0 | 3.1 | 5.9 | 3.2 | 11.2 | 2.6 | 15.5 |
| PSI | gamma | 24 | 7.6 | 5.6 | 6.8 | 1.0 | 16.0 | 0.3 | 20.1 |
| SR | offset | 25 | 5.0 | 2.9 | 5.0 | 0.9 | 7.2 | 0.3 | 13.4 |
| PSI | offset | 24 | 6.9 | 4.7 | 6.3 | 1.5 | 13.4 | 0.9 | 18.5 |

| Preoperative absolute deviations after correction in PSI group | | | | | | | | | |
| --- | --- | --- | --- | --- | --- | --- | --- | --- | --- |
| group | variable | N | mean | sd | median | p10 | p90 | min | max |
| SR | alpha | 25 | 4.5 | 4.4 | 2.4 | 0.9 | 10.1 | 0.6 | 17.3 |
| PSI | alpha | 24 | 4.2 | 3.0 | 3.8 | 1.1 | 8.8 | 0.0 | 10.1 |
| SR | beta | 25 | 4.7 | 4.3 | 3.2 | 0.9 | 11.4 | 0.7 | 17.0 |
| PSI | beta | 24 | 5.6 | 4.8 | 4.3 | 0.8 | 10.5 | 0.2 | 22.3 |
| SR | gamma | 25 | 7.0 | 3.1 | 5.9 | 3.2 | 11.2 | 2.6 | 15.5 |
| PSI | gamma | 24 | 8.0 | 5.2 | 7.7 | 1.9 | 16.2 | 1.3 | 20.6 |

| Postoperatively measured values | | | | | | | | | |
| --- | --- | --- | --- | --- | --- | --- | --- | --- | --- |
| group | variable | N | mean | sd | median | p10 | p90 | min | max |
| SR | alpha | 25 | 88.9 | 3.3 | 88.7 | 85.2 | 93.1 | 81.8 | 94.2 |
| PSI | alpha | 24 | 89.0 | 2.3 | 89.1 | 86.3 | 92.9 | 84.8 | 94.5 |
| SR | beta | 25 | 88.8 | 3.4 | 88.7 | 83.7 | 93.2 | 81.7 | 94.2 |
| PSI | beta | 24 | 89.2 | 2.5 | 89.0 | 86.2 | 93.1 | 84.9 | 95.4 |
| SR | gamma | 25 | 89.8 | 2.4 | 89.8 | 86.4 | 92.7 | 85.6 | 94.1 |
| PSI | gamma | 24 | 88.7 | 2.8 | 88.5 | 85.4 | 93.1 | 83.2 | 94.1 |
| SR | offset | 25 | 2.3 | 2.0 | 2.3 | -0.4 | 4.2 | -3.2 | 5.2 |
| PSI | offset | 24 | 2.6 | 2.8 | 2.9 | -1.0 | 6.2 | -2.2 | 7.4 |

| Postoperatively measured values after correction in PSI group | | | | | | | | | |
| --- | --- | --- | --- | --- | --- | --- | --- | --- | --- |
| group | variable | N | mean | sd | median | p10 | p90 | min | max |
| SR | alpha | 25 | 88.9 | 3.3 | 88.7 | 85.2 | 93.1 | 81.8 | 94.2 |
| PSI | alpha | 24 | 88.1 | 2.5 | 88.5 | 85.1 | 91.7 | 83.0 | 92.0 |
| SR | beta | 25 | 88.8 | 3.4 | 88.7 | 83.7 | 93.2 | 81.7 | 94.2 |
| PSI | beta | 24 | 88.3 | 2.5 | 88.4 | 84.8 | 91.9 | 83.5 | 92.4 |
| SR | gamma | 25 | 89.8 | 2.4 | 89.8 | 86.4 | 92.7 | 85.6 | 94.1 |
| PSI | gamma | 24 | 88.4 | 2.4 | 88.2 | 85.4 | 91.6 | 84.4 | 93.2 |

| Postperative deviations | | | | | | | | | |
| --- | --- | --- | --- | --- | --- | --- | --- | --- | --- |
| group | variable | N | mean | sd | median | p10 | p90 | min | max |
| SR | alpha | 25 | -1.1 | 3.3 | -1.3 | -4.8 | 3.1 | -8.2 | 4.2 |
| PSI | alpha | 24 | -1.0 | 2.3 | -0.9 | -3.7 | 2.9 | -5.2 | 4.5 |
| SR | beta | 25 | -1.2 | 3.4 | -1.3 | -6.3 | 3.2 | -8.3 | 4.2 |
| PSI | beta | 24 | -0.8 | 2.5 | -1.0 | -3.8 | 3.1 | -5.1 | 5.4 |
| SR | gamma | 25 | -0.2 | 2.4 | -0.2 | -3.6 | 2.7 | -4.4 | 4.1 |
| PSI | gamma | 24 | -1.3 | 2.8 | -1.5 | -4.6 | 3.1 | -6.8 | 4.1 |

| Postperative deviations after correction in PSI group | | | | | | | | | |
| --- | --- | --- | --- | --- | --- | --- | --- | --- | --- |
| group | variable | N | mean | sd | median | p10 | p90 | min | max |
| SR | alpha | 25 | -1.1 | 3.3 | -1.3 | -4.8 | 3.1 | -8.2 | 4.2 |
| PSI | alpha | 24 | -1.9 | 2.5 | -1.5 | -4.9 | 1.7 | -7.0 | 2.0 |
| SR | beta | 25 | -1.2 | 3.4 | -1.3 | -6.3 | 3.2 | -8.3 | 4.2 |
| PSI | beta | 24 | -1.7 | 2.5 | -1.6 | -5.2 | 1.9 | -6.5 | 2.4 |
| SR | gamma | 25 | -0.2 | 2.4 | -0.2 | -3.6 | 2.7 | -4.4 | 4.1 |
| PSI | gamma | 24 | -1.6 | 2.4 | -1.8 | -4.6 | 1.6 | -5.6 | 3.2 |

| Postoperative absolute deviations | | | | | | | | | |
| --- | --- | --- | --- | --- | --- | --- | --- | --- | --- |
| group | variable | N | mean | sd | median | p10 | p90 | min | max |
| SR | alpha | 25 | 2.7 | 2.0 | 2.1 | 0.6 | 4.8 | 0.2 | 8.2 |
| PSI | alpha | 24 | 2.0 | 1.5 | 1.6 | 0.4 | 4.5 | 0.2 | 5.2 |
| SR | beta | 25 | 2.9 | 2.2 | 2.3 | 0.5 | 6.3 | 0.2 | 8.3 |
| PSI | beta | 24 | 2.1 | 1.6 | 1.6 | 0.2 | 3.9 | 0.1 | 5.4 |
| SR | gamma | 25 | 2.0 | 1.3 | 1.5 | 0.6 | 3.9 | 0.2 | 4.4 |
| PSI | gamma | 24 | 2.5 | 1.7 | 2.2 | 0.2 | 4.6 | 0.1 | 6.8 |
| SR | offset | 25 | 2.6 | 1.5 | 3.0 | 0.6 | 4.2 | 0.4 | 5.2 |
| PSI | offset | 24 | 3.1 | 2.2 | 2.9 | 0.6 | 6.2 | 0.1 | 7.4 |

| Postoperative absolute deviations after correction in PSI group | | | | | | | | | |
| --- | --- | --- | --- | --- | --- | --- | --- | --- | --- |
| group | variable | N | mean | sd | median | p10 | p90 | min | max |
| SR | alpha | 25 | 2.7 | 2.0 | 2.1 | 0.6 | 4.8 | 0.2 | 8.2 |
| PSI | alpha | 24 | 2.4 | 1.9 | 1.8 | 0.6 | 4.9 | 0.3 | 7.0 |
| SR | beta | 25 | 2.9 | 2.2 | 2.3 | 0.5 | 6.3 | 0.2 | 8.3 |
| PSI | beta | 24 | 2.5 | 1.7 | 2.0 | 0.6 | 5.2 | 0.3 | 6.5 |
| SR | gamma | 25 | 2.0 | 1.3 | 1.5 | 0.6 | 3.9 | 0.2 | 4.4 |
| PSI | gamma | 24 | 2.3 | 1.6 | 2.5 | 0.2 | 4.6 | 0.1 | 5.6 |

sd: standard deviation; p10:10%ile; p90 :90%ile ; min : minimum ; max : maximum
