# Supplementary material for: Patient-Specific Instrumentation vs Standard Referencing in Total Ankle Arthroplasty: A Comparison of the Radiologic Outcome
Source: Foot Ankle Int. 2022 Feb 24;43(6):741–9. doi: 10.1177/10711007221077100 (PMC9168897; doi:10.1177/10711007221077100)
Supplement: sj-docx-2-fai-10.1177_10711007221077100 – Supplemental material for Patient-Specific Instrumentation vs Standard Referencing in Total Ankle Arthroplasty: A Comparison of the Radiologic Outcome [file sj-docx-2-fai-10.1177_10711007221077100.docx]

Supplementary Table 2: Distributional characteristics of the differences between the two observers

|  | Variable | Difference | | | | | Absolute difference | | | ICC |
| --- | --- | --- | --- | --- | --- | --- | --- | --- | --- | --- |
|  |  | mean | sd | p5 | median | p95 | mean | med | p95 |  |
| Pre-operative images | alpha | -0.31 | 5.63 | -9.90 | 0.0 | 8.8 | 4.16 | 4.0 | 10.3 | 0.59 |
|  | beta | 0.02 | 6.31 | -12.80 | 0.0 | 10.0 | 4.41 | 2.4 | 13.3 | 0.63 |
|  | gamma | -1.83 | 3.63 | -7.90 | -0.7 | 3.1 | 3.04 | 2.0 | 7.9 | 0.73 |
|  | offset | -0.66 | 2.08 | -3.60 | -0.4 | 1.3 | 1.28 | 0.8 | 5.5 | 0.86 |
| Post-operative images | alpha | 0.28 | 1.34 | -2.10 | 0.2 | 2.6 | 0.97 | 0.6 | 2.6 | 0.89 |
|  | beta | -0.20 | 1.44 | -2.60 | -0.3 | 2.5 | 0.99 | 0.7 | 3.2 | 0.89 |
|  | gamma | -0.10 | 0.57 | -1.10 | 0.0 | 0.8 | 0.46 | 0.4 | 1.2 | 0.98 |
|  | offset | 0.11 | 0.84 | -1.20 | 0.1 | 1.4 | 0.66 | 0.6 | 1.6 | 0.90 |

sd: standard deviation; p5: 5%ile; p95: 95%ile; ICC: intra-class correlation coefficient
