# Supplementary material for: Patient-Specific Instrumentation vs Standard Referencing in Total Ankle Arthroplasty: A Comparison of the Radiologic Outcome
Source: Foot Ankle Int. 2022 Feb 24;43(6):741–9. doi: 10.1177/10711007221077100 (PMC9168897; doi:10.1177/10711007221077100)
Supplement: sj-docx-3-fai-10.1177_10711007221077100 – Supplemental material for Patient-Specific Instrumentation vs Standard Referencing in Total Ankle Arthroplasty: A Comparison of the Radiologic Outcome [file sj-docx-3-fai-10.1177_10711007221077100.docx]

Table 3: Absolute deviations from the intended alignment classified into groups. Shown are absolute and relative frequencies.

|  |  | <3° | 3°-5° | >5° |
| --- | --- | --- | --- | --- |
| alpha | SR | 15/60% | 8/32% | 2/8% |
|  | PSI | 15/62% | 7/29% | 2/8% |
| beta | SR | 15/60% | 7/28% | 3/12% |
|  | PSI | 16/67% | 5/21% | 3/12% |
| gamma | SR | 18/72% | 7/28% | 0/0% |
|  | PSI | 16/67% | 6/25% | 2/8% |
