# Supplementary material for: Patient-Specific Instrumentation vs Standard Referencing in Total Ankle Arthroplasty: A Comparison of the Radiologic Outcome
Source: Foot Ankle Int. 2022 Feb 24;43(6):741–9. doi: 10.1177/10711007221077100 (PMC9168897; doi:10.1177/10711007221077100)
Supplement: sj-docx-4-fai-10.1177_10711007221077100 – Supplemental material for Patient-Specific Instrumentation vs Standard Referencing in Total Ankle Arthroplasty: A Comparison of the Radiologic Outcome [file sj-docx-4-fai-10.1177_10711007221077100.docx]

Table 4: Implant size predicted by PSI and actual implant size used. Shown are absolute frequencies. Agreement between predicted and actual size is marked in bold.

| Tibia | | | |
| --- | --- | --- | --- |
| Predicted size | Actual size used | | |
|  | 3 | 4 | 5 |
| 3 | **4** | 1 | 0 |
| 4 | 1 | **6** | 1 |
| 5 | 0 | 0 | **11** |

| Talus | | | | |
| --- | --- | --- | --- | --- |
| Predicted size | Actual size used | | | |
|  | 2 | 3 | 4 | 5 |
| 2 | **0** | 0 | 0 | 0 |
| 3 | 1 | **6** | 0 | 0 |
| 4 | 0 | 0 | **6** | 1 |
| 5 | 0 | 0 | 6 | **4** |
